# Potential benefits of synchronous action observation and motor imagery: a commentary on Eaves et al. 2022

**DOI:** 10.1007/s00426-023-01895-9

**Published:** 2023-11-10

**Authors:** Shaheed Azaad, Natalie Sebanz

**Affiliations:** Central European University, Vienna, Austria

## Abstract

In a recent *Psychological Research* article, Eaves et al. (2022) review the literature on how motor imagery (MI) practice combined with action observation (AO) enhances motor performance. The authors propose that the synchronous form of AO and MI (AOMI) affords unique benefits to performance that are not possible when the two interventions are performed asynchronously. We discuss three questions raised by Eaves et al.’s review: (1) are there any clear advantages to synchronous AOMI? (2) Are there super-additive benefits to AOMI, and if so, are they unique to synchronous AOMI? (3) How might coordinative AOMI, in which people imagine complementary actions, facilitate joint actions?

In their recent article, Eaves et al. (2022) provide an insightful overview of the literature on how action observation (AO) interacts with motor imagery (MI). Their focus is on how synchronous AO and MI (sAOMI) can enhance the practice and performance of motor actions. In this commentary, we highlight and discuss three questions raised by their review: (1) is there any clear advantage to AO and MI being performed synchronously vs asynchronously? (2) are the benefits of combining AO and MI super-additive, and are super-additive effects specific to sAOMI? (3) how could “coordinative AOMI”, where the observed and the imagined action are complementary rather than the same, enhance the performance of joint actions?

## Evidence for sAOMI benefits over aAOMI

Motor imagery practice has been shown to improve performance in both action learning and rehabilitation contexts (Ladda et al., 2021 for a review). Although there is clear evidence that adding AO to MI practice (or AOMI) further enhances performance, how these two interventions are best combined remains an open question.

In their review, Eaves et al. distinguish between two types of AOMI: in *synchronous* AOMI (sAOMI), MI occurs during AO; while in *asynchronous* AOMI (aAOMI), AO either precedes or alternates with MI. The authors argue that sAOMI is ‘unique’ in that performing AO and MI synchronously might enable them to interact and provide a ‘super-additive’ benefit to performance. Specifically, sAOMI might draw attention to the spatiotemporal characteristics of an action. This enables observers to refine and update their internal simulations according to the seen action in real-time. Further, Eaves et al. propose that AO and MI might complement each other—while AO promotes effector-specific encoding of motor programs, MI serves to acquire global movement features, such as rhythmic timing.

It is unclear whether sAOMI indeed improves performance beyond aAOMI. Eaves et al. demonstrate that sAOMI practice produces performance benefits beyond those possible from either AO or MI alone. However, as the authors note, the few studies that directly compare the two forms of AOMI practice fail to find a difference between them in subsequent performance. Research on neurotypical participants has shown that aAOMI improves performance either as much as (Romano Smith et al., 2019; Romano-Smith et al., 2018) or more than (Lin et al., 2022), sAOMI. In sum, there is little empirical evidence for the proposal that AO and MI interact uniquely in synchronous versus asynchronous AOMI.^1^ Future research is needed to determine under which conditions, if any, sAOMI has an advantage over aAOMI. As we will discuss, the practice of joint actions involving the coordination of complementary motor programs constitutes one domain where sAOMI might truly trump aAOMI.

## Super-additive effects of sAOMI

Eaves et al. propose that we can test for super-additive effects of AO on MI by contrasting performance benefits between aAOMI and sAOMI practice. However, this test assumes that any super-additive effects must occur in real time. Another possibility is that AO could augment subsequent and concurrent MI equally and thus produce super-additive effects that we could not detect by directly contrasting the two types of AOMI. Instead, if we assume that AO augments MI (and not vice versa), the critical test for an interaction between these interventions would be to compare AO followed by MI to MI followed by AO. While AO could enhance subsequent MI practice, this is not possible if MI occurs first. Therefore, if AO and MI interact, we would expect AOMI to yield greater performance benefits than MI followed by AO, in which the combined benefit could only be additive.

It seems an important goal for future research to establish whether super-additive effects of AO and MI can be found for aAOMI. By which mechanisms could AO and MI have super-additive effects that do not rely on them co-occurring? As mentioned by Eaves et al., one possibility is that AO enhances the fidelity of subsequent MI. Considering that AO training improves the ease with which people can engage in MI (Wright et al., 2015), and that MI practice benefits depend upon one’s imagery ability (Robin et al., 2007), AO might help us generate more precise MI, which in turn would benefit action performance.

Furthermore, motor learning studies have shown that people learn best when they focus on the outcomes of their actions rather than the specific movements required to achieve them (Wulf, 2013 for a review). Recently, Bach et al. (2022) proposed an effect-based account of MI practice, in which MI involves imagining desired action outcomes rather than simulating particular movements. If MI relies on effect imagery, AO might enhance MI practice by clarifying the relationship between movements and the intended outcomes. This could be especially true for actions where the very early stages of motor planning depend upon a desired end-state (Bhoyroo et al., 2019; Rosenbaum et al., 1996). In these cases, seeing the end-state beforehand might help an observer refine MI more than seeing the action concurrently. Such a mechanism might explain why research has found an advantage for aAOMI for more kinematically complex actions (golf putting; Lin et al., 2022) but not for simpler ones (dart throwing; Romano-Smith et al., 2018).

Given the possibility of super-additive effects of aAOMI, the question arises in what ways sAOMI might produce benefits beyond aAOMI or are unique to sAOMI. One possibility is that actions that require precise timing, like playing music, benefit from online temporal refinement afforded uniquely by sAOMI. On the one hand, sAOMI might enable entrainment (Clayton et al., 2020; Phillips-Silver & Keller, 2012) between imagined and observed actions which translates to higher temporal fidelity in subsequent performance. On the other hand, as Eaves et al. also suggest, sAOMI could help observers identify and correct subtle timing discrepancies between imagined and observed actions that would be otherwise difficult to detect.

## Coordinative AOMI and joint action performance

An intriguing possibility raised by Eaves et al. is that sAOMI might be especially helpful when people practice performing part of a joint action. Observing the actions of another individual and concurrently imagining performing complementary actions could yield practice benefits that aAOMI does not afford. Many joint actions depend more on the spatiotemporal *relationship* between multiple people’s actions than their individual contributions (Sebanz & Knoblich, 2021). In these cases, sAOMI might help us form better predictive models of the timing of others’ actions relative to our own (Wolf et al., 2018) and, in turn, refine the spatiotemporal characteristics of our MI for smoother coordination.

Future studies using coordinative AOMI could also test the prediction that the variability of complementary actions in AO facilitates learning. As Eaves et al. point out, the importance of variability in individual practice is well established. Recent studies on joint action suggest that interacting with variable partners can improve individual and joint performance (Ivanova et al., 2022; Lev-Ari & Sebanz, 2020; Sabu et al., 2020). This raises the possibility that coordinative AOMI with variable partners could be used to generate substantial learning benefits.

## Conclusion

In sum, whether the benefits of combining AO and MI are merely additive or super-additive remains an open question. To test whether AO augments MI, future research might compare the benefits of AOMI with MI followed by AO, which would afford additive benefits but not any effect of AO on MI. Studies that systematically manipulate the temporal complexity of observed actions could shed further light on whether AO and MI interact in a way unique to sAOMI. Finally, coordinative AOMI appears to be a promising new approach both for improving our understanding of joint action learning and for developing training programs that draw on partner variability. By exploring in depth how action observation and motor imagery might interact, Eaves et al. have provided us with important and challenging questions for future research.

## Data Availability

Not applicable.

## Footnotes

1 While there is evidence from Sun et al. (2016) for an advantage of sAOMI in stroke patients, the study’s outcome measures were related to more general motor ability rather than performance of a mentally-practiced action. Additionally, as Romano et al. (2018) suggest, sAOMI might be advantageous for stroke patients due to them experiencing impairments in working memory that make it challenging to remember observed actions for subsequent MI.
